# P-2049. Detecting Staphylococcus aureus Bacteremia in Patients with S. aureus Bacteriuria: A Multicenter Quality Improvement Project

**DOI:** 10.1093/ofid/ofaf695.2213

**Published:** 2026-01-11

**Authors:** Timothy J Whitman, W Kemper Alston, Jennifer Beveridge, Campbell Brendan, Keith Collins, Juvena Hitt, Jessie Leyse, Allen B Repp

**Affiliations:** Larner College of Medicine, University of Vermont Health Network, Shelburne, VT; University of Vermont Medical Center, Burlington, VT; Larner College of Medicine, University of Vermont Health Network, Plattsburg, New York; Larner College of Medicine, University of Vermont Health Network, Shelburne, VT; Larner College of Medicine, University of Vermont Health Network, Shelburne, VT; University of Vermont Health Network, Burlington, Vermont; Larner College of Medicine, University of Vermont Health Network, Shelburne, VT; Larner College of Medicine, University of Vermont Health Network, Shelburne, VT

## Abstract

**Background:**

*Staphylococcus aureus* bacteriuria (SABU) may indicate *Staphylococcus aureus* bacteremia (SAB) but this connection is not well defined. Identifying patients with SABU at risk for SAB may promote early detection and treatment of SAB.

**Methods:**

We conducted a multicenter quality improvement (QI) project to detect SAB among patients with SABU across six hospitals within the University of Vermont Health Network (UVMHN), which provides care for approximately 1 million patients in Vermont and upstate New York. Infectious Diseases (ID) specialists screened records of all UVMHN patients with SABU weekly and contacted ordering provider teams if they felt blood cultures, other diagnostic tests, or treatment changes were warranted. ID specialists documented risk factors for both SABU and concomitant SAB and reevaluated patient records 30 days later to capture outcomes.

**Results:**

From Sept 2024-April 2025, we screened a total of 115 patients with SABU, averaging 5 patients per week. The screening process took less than 10 minutes/patient. The mean age was 64 years and 57% were male. MSSA was detected in 70% of urine cultures and MRSA in 29%. We found no cases of undiagnosed SAB, and all 11 (10%) patients with concomitant SABU and SAB had been identified by the ordering team. Injection drug use (IDU) was more common in patients with SAB than those with SABU only (27% vs 2%, p< 0.01). We observed a 27% 30-day mortality rate in patients with SAB compared to 5% in SABU only (p< 0.01).

**Conclusion:**

We found 10% of patients with SABU had SAB. Patients with SAB were more likely to have IDU and had a markedly higher 30-day mortality than patients with SABU only. Although the screening process was efficient, we did not identify undiagnosed SAB. Reviewing all patients with SABU may not be justified and future efforts to identify risk factors for SAB among patients SABU could help focus these interventions.

**Disclosures:**

All Authors: No reported disclosures

